# Phase Composition of Al-Si Coating from the Initial State to the Hot-Stamped Condition

**DOI:** 10.3390/ma14051125

**Published:** 2021-02-27

**Authors:** Vojtech Kucera, Marcello Cabibbo, Filip Prusa, Jaroslav Fojt, Jaroslav Petr-Soini, Tomas Pilvousek, Marie Kolarikova, Dalibor Vojtech

**Affiliations:** 1 Department of Metals and Corrosion Engineering, University of Chemistry and Technology, Prague, Technická 5, 166 28 Prague 6, Czech Republic; prusaf@vscht.cz (F.P.); fojtj@vscht.cz (J.F.); vojtechd@vscht.cz (D.V.); 2 Press Shop and Welding Shop Technical Service, ŠKODA AUTO a.s., tř. Václava Klementa 869, 293 01 Mladá Boleslav, Czech Republic; tomas.pilvousek@skoda-auto.cz; 3 Department of Industrial Engineering and Mathematical Sciences (DIISM), Università Politecnica delle Marche, Via Brecce Bianche, 60131 Ancona, Italy; m.cabibbo@staff.univpm.it; 4 Department of Materials Engineering, Czech Technical University in Prague, Karlovo náměstí 13, 121 35 Prague 2, Czech Republic; jaroslav.petr@fs.cvut.cz; 5 Department of Manufacturing Technology, Czech Technical University in Prague, Technická 4, 166 07 Prague 6, Czech Republic; Marie.Kolarikova@fs.cvut.cz

**Keywords:** austenitization, die-quenching, 22MnB5 steel, Al-Si coating, TEM-SAED

## Abstract

The chemical and phase composition of the coating and the coating/substrate interface of an Al-Si-coated 22MnB5 hot stamped steel was investigated by means of SEM-EDS, XRD, micro-XRD and electron diffraction. Moreover, the surface profile was analyzed by XPS and roughness measurements. The XPS measurements showed that the thickness of the Si and Al oxide layers increased from 14 to 76 nm after die-quenching, and that the surface roughness increased as well as a result of volume changes caused by phase transformations. In addition to the FeAl(Si) and Fe_2_Al_5_ phases and the interdiffusion layer forming complex structures in the coating, electron diffraction confirmed the presence of an Fe_2_Al_5_ phase, and also revealed very thin layers of Fe_3_(Al,Si)C, Fe_2_(Al,Si)_5_ and Al-bearing rod-shaped particles in the immediate vicinity of the steel interface. Moreover, the scattered nonuniform layer of the Fe_2_Al_8_Si phase was identified in the outermost layer of the coating. Despite numerous studies devoted to researching the phase composition of the Al-Si coating applied to hot stamped steel, electron diffraction revealed very thin layers and particles on the coating/substrate interface and outermost layer, which have not been analyzed in detail.

## 1. Introduction

With incessantly increasing pressure on the automotive industry, manufacturers are being forced to develop new technologies and materials to reduce car body weight. This can be achieved by hot stamping technology capable of producing press-hardened steel (PHS), reaching an ultimate tensile strength of up to 2000 MPa. Hot stamping significantly suppresses the spring-back effect compared to conventional cold press forming, and ensures the geometric accuracy of pressed parts. PHS is used mostly to produce safety components such as A-pillars, B-pillars, bumpers, roof rails, rocker rails and tunnels. Due to its extremely high strength, PHS enables sheet thickness reduction and helps to fulfill the constantly stricter standards of CO_2_ emissions. Moreover, PHS enhances the toughness of the vehicle frame and passenger safety. Therefore, its proportional content in car bodies attracts increasing attention [1,2,3].

Hot stamping technology offers two different variants of processing: (i) direct hot stamping, and (ii) indirect hot stamping. In both variants, the input material is low-alloyed steel with a ferritic/pearlitic microstructure and a strength of approximately 600 MPa [2]. In direct hot stamping, the sheet blank is heated to temperatures of 850–950 °C and held for 3–10 min to obtain a fully austenitic microstructure with a low tensile strength and high ductility (~200 MPa, ~40% total elongation) [4,5,6,7,8]. After subsequent transferral into the water-cooled press and a temperature reduction to 600–800 °C, the blank is formed and hardened in a single step. The cooling rate should be at least 25–27 K/s to cool the steel to approximately 200 °C (M_f_—martensite finish point) [2,4].

On the other hand, during the indirect process, the final shape of the press part is nearly attained by the cold forming of ferritic/pearlitic steel, which is subsequently austenized and followed only by die-quenching and calibration in the water-cooled press. However, in both processes, during austenitization, the steel is exposed to high temperatures in the furnace, causing the simultaneous decarburization and oxidation of the steel surface. As such, protective coatings are applied to the steel to prevent this [2,4,7,9].

Zinc is applied to most parts of the car body. However, the Zn coating is difficult to process by direct hot stamping because the Fe-Zn couple tends to experience liquid metal embrittlement during deformation. Indirect hot stamping enables the processing of Zn coatings by avoiding high levels of deformation at elevated temperatures, although it is more demanding on the equipment, and thus more expensive [10]. Therefore, the Al-Si-coated press-hardened steel is most widespread in the automotive industry. Its advantage is that the Al-Si coating can sustain high temperatures and protects the steel substrate from oxidation and decarburization by forming thermodynamically stable oxides rich in aluminum and silicon [2,4,5,9]. Moreover, in contrast to the Zn coating, the Al-Si coating does not suffer from liquid metal embrittlement during direct hot stamping, and further surface treatment, such as sandblasting or dry ice cleaning to remove the oxide layer, is not applied for the Al-Si coating compared to the Zn coating [11]. However, high temperatures together with die-quenching result in the occurrence of inhomogeneities and the forming of voids and cracks in the coatings. As such, with the development of novel methods such as electron beam and ion beam irradiation or mechanical surface treatments, the surface properties of PHS could be improved even at an industrial scale. The beneficial effect of Zr^+^ ion beam irradiation on fatigue life was shown in Ref. [12]. The formation of a nanostructured surface layer consisting of ultra-fine zirconates particles as well as a thermally softened layer by irradiation annealing led to improved fatigue behavior. Electron beam surface treatment causing superfast heating and cooling, as used in Ref. [13], led to the submicron dendrite structure in the topmost layer of the surface, and thermoelastic stresses resulting in increased durability during multicycle fatigue loading. Wang et al. [14] combined ultrasonic surface rolling, a method of severe plastic deformation, with electro-pulsing to improve the wear resistance and hardness of the coating. The authors also observed the healing of surface cracks.

PHS is commonly aluminized by hot-dipping into a bath containing 7–11 wt.% Si, up to 3 wt.% Fe and Al bulk at temperatures of approximately 670–700 °C [4]. Si is added to the bath to form an inhibition layer of the Fe_2_Al_8_Si (τ_5_) phase (also referred to in the literature as Fe_2_Al_7_Si and Fe_2_Al_7.4_Si), retarding the formation of the very brittle Fe_2_Al_5_ phase while significantly influencing the formation of a layered structure of the coating [15,16,17,18]. However, the melting temperature of the Al-Si coating with a typical chemical composition of Al-Si10 is approximately 600 °C, which is substantially lower than the austenitization temperature (~920 °C) [2,4]. Therefore, during the holding time, the Al-Si coating melts. The melting of the Al-Si coating further enhances the mutual diffusion-related effects between the steel and the liquid metal coating. The iron enrichment increases the melting temperature via the formation of binary Al-Fe or ternary Al-Fe-Si phases. These phases expand from the substrate/coating interface to the top surface layer, and the melt gradually solidifies, increasing the coating thickness. The final structure of the coating after hot stamping consists of several layers with different amounts of Fe [2,4,6]. The chemical composition and phase distribution of the Al-Si coatings are strongly dependent on the heating and transport conditions. Moreover, the multiphase structure is also connected to the occurrence of pores and cracks in the coating leading up to the steel substrate [2,6,19]. Windmann et al. [6,17] identified the layers in the coating at the substrate/coating interface as follows: the interdiffusion layer was formed by the solid solution α-Fe(Al,Si) adjacent to the FeAl continuous layer, and the matrix of the coating consisting of Fe_2_Al_5_ and the FeAl phase forming a continuous layer or separate islands was embedded in the Fe_2_Al_5_ matrix. Gui et al. [20] and Liang et al. [21], using very similar processing conditions, also observed an α-Fe(Al,Si) interdiffusion layer and the FeAl phase was dispersed, although in the Fe_2_Al matrix of the coating. Furthermore, the continuous layer or island-like phase, denoted as FeAl in previous works, was described as Fe_2_SiAl_2_ (τ_1_) and Fe_2_Si_2_Al_5_ (τ_2_) in Refs. [5,22,23], with an interdiffusion layer formed of Fe_3_Al [5,23]. The most recent work from Cho et al. [24] stated that the three-phase interdiffusion layer consisted of α-Fe(Al), Fe_3_Al, FeAl, the matrix of Fe_2_Al_5_, and a continuous layer or island-like phase distributed in the matrix as a mixture of FeAl and an unidentified Fe_x_Al_x_Si_z_ ternary phase.

Despite these numerous studies of the coating, the findings of the resulting phase composition, coating/substrate interface, and void distribution in the coating are inconsistent. This discrepancy might be caused by the presence of non-stoichiometric phases in the Al-Fe system with different solubilities of Si and a rather high intricacy of Al-Fe and Al-Fe-Si systems. Therefore, SEM-EDS and XRD analyses might struggle to identify the phase composition and the presence of fine layers and particles. Moreover, a detailed TEM analysis of the coating/substrate interface and phases across the coating is still missing, to our best knowledge. As such, this work is focused on the TEM-SAED analysis of the coating/substrate interface, and the description of phases through the coating, as well as an analysis of the top surface layer by means of XPS and roughness measurements to increase the general knowledge of Al-Si coatings.

## 2. Materials and Methods

Commercially available manganese-boron steel (22MnB5) with a ferritic/pearlitic microstructure and Al-Si hot-dipped coating was used in the present study. The steel sheet had a thickness of 1.5 mm and was double-side-coated (150 g/m^2^). The Al-Si coating was commercially produced by continuous immersion in a bath with an approximate chemical composition of 90 wt.% Al and 10 wt.% Si. The as-received steel sheets with dimensions of 200 × 300 mm were first cut into smaller specimens (80 × 40 mm) for further investigation. The chemical composition of the as-received steel (Table 1) was measured with an optical emission spectrometry (OES) system (Bruker G8 Galileo, Billerica, Massachusetts, MA, USA). The specimens were heated up to a temperature of 920 °C in an electrical resistance furnace (Laboratorní pece Martínek, Kladno, Czech Republic) with an 8 min dwell time, close to the industry conditions. After that, the specimens were transported (2 s) and die-quenched to achieve martensitic transformation without any imposed deformation. The steel die was sufficiently robust to achieve the martensitic transformation and was air-cooled. The samples used for microstructural observation were prepared by a standard metallography procedure, which included grinding the samples with SiC abrasive papers (P320–P2000), polishing with polycrystalline diamond suspensions (9–1 µm), and etching the samples in 5% nital [25]. The microstructure was observed using an optical microscope (Olympus PME-3, Olympus, Tokyo, Japan) and a scanning electron microscope (Tescan Vega 3 LMU, 20 kV, SE + BSE detectors, TESCAN, Brno, Czech Republic) equipped with an energy dispersive spectroscopy detector (Oxford Instruments INCA 350, 20 mm^2^, Oxford Instruments, Abingdon, England). For further phase analysis, X-ray diffraction data were collected at room temperature with an X’Pert3 Powder θ-θ powder diffractometer (PANanalytical, Almelo, Netherlands) with a parafocusing Bragg-Brentano geometry using CuK_α_ radiation (λ = 0.15418 nm, U = 40 kV, I = 30 mA). The micro-X-ray diffraction data were obtained by a D8 Discover microdiffractometer (Bruker, Billerica, Massachusetts, MA, USA) with a 0.1 mm collimator at room temperature in a parallel geometry using the wavelength of CoK_α_ radiation (λ = 0.27903 nm, U = 35 kV, I = 40 mA). Two Debye-Scherrer frames were obtained in the θ-θ geometry for 2θ = 26° and 56° with a VÅNTEC-500 2D detector (Bruker, Billerica, Massachusetts, MA, USA) and a measurement time of 30 min per frame. The thicknesses of the coatings and interfacial layers in the hot-dipped condition and after austenitization and die-quenching were measured by image analysis using ImageJ software 1.8.0_172. The measured thickness is an average value from at least 60 measurements.

The coating surface of the as-received 22MnB5 in the hot-dipped condition and after die-quenching was studied using an X-ray photoelectron spectroscopy (XPS) system (ESCAprobe P, Omicron Nanotechnology Ltd., Taunusstein, Germany) equipped with an Al K_α_ (λ = 1486.7 eV) X-ray source. The spectra were measured with an energy step size of 0.05 eV and normalized to the binding energy of the C1s peak (285.0 eV). The data used for the chemical state evaluation were obtained from the NIST X-ray Photoelectron Spectroscopy Database. The roughness of the coated samples was measured with a distance of 17.5 mm, a cutoff of 2.5 mm, and a 2 µm sensor tip, and a Keyence VHX-7000 Series digital microscope (Keyence, Osaka, Japan) was used for the roughness measurements and surface topology profile.

TEM observations were carried out by using a Philips Inc. CM-20^®^ microscope (Amsterdam, The Netherlands) operated at 200 kV with a double tilt specimen holder equipped with a liquid nitrogen cooling stage. TEM discs were prepared by cross-sectioning the two sections together that were embedded in an alcohol-based resin. The two sections were embedded in a 2 mm inner diameter pure copper tube with a thickness of 1 mm. This wafer material was cut to a thickness of 200 µm, grinded, and polished down to 100–90 µm. Then, this material was dimpled on both sides to obtain a final central thickness ranging from 20 to 25 µm. The final preparation method used to achieve electron transparency was performed by a Gatan Inc. precision ion polishing system (PIPS) (Pleasanton, CA, USA) with double Ar^+^ flux set at an initial incident angle of 8°, which was operated for a few minutes, and then the angle was changed to 6° and finally 4°. PIPS was performed for 2 h, and the sample was kept cool by flowing liquid nitrogen beneath the sample stage. Phase identification was performed by TEM by indexing the selected area electron diffraction (SAEDP) patterns. To properly obtain SAEDPs from the small particles and small areas, a converged beam (CB) was used.

## 3. Results and Discussion

### 3.1. Chemical and Phase Composition of the As-Received 22MnB5 in the Hot-Dipped Condition

**Figure 1:**
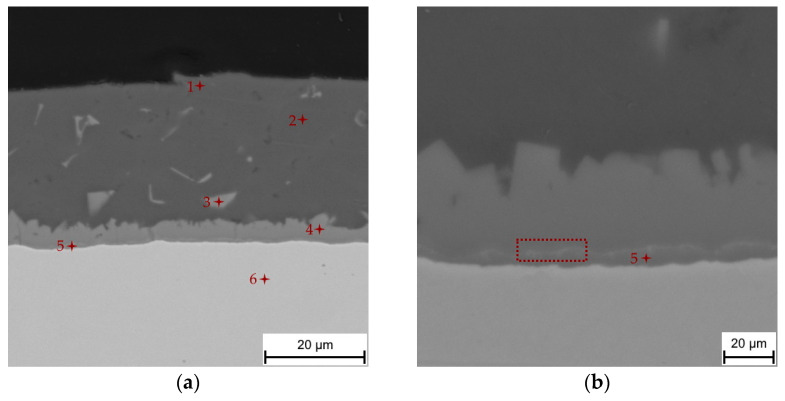
Scanning electron microscope (SEM) micrographs of (**a**) the Al-Si hot-dipped coating on as-received 22MnB5 boron steel and (**b**) a detailed image of the steel/coating interface.

**Figure 2:**
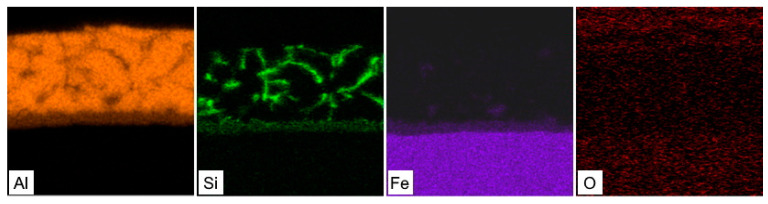
SEM-EDS elemental distribution maps of the Al-Si hot-dipped coating on as-received 22MnB5 boron steel.

**Figure 3:**
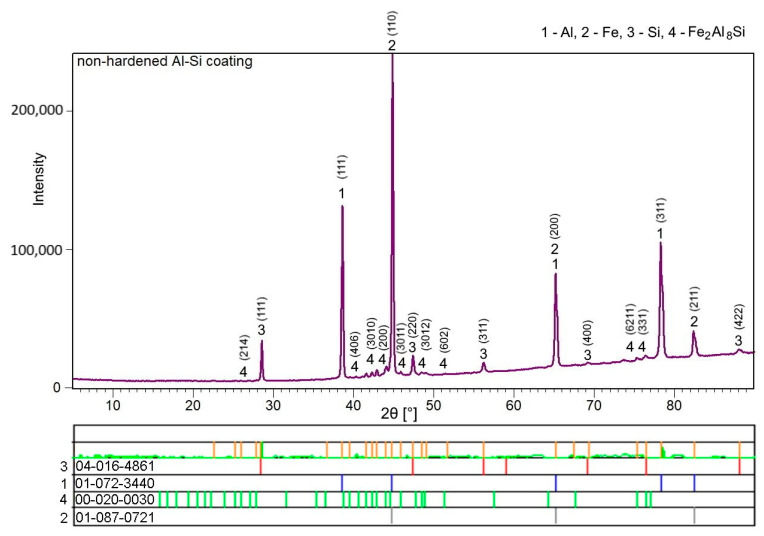
XRD pattern of the Al-Si hot-dipped coating on as-received 22MnB5 boron steel.

The hot-dipped Al-Si coating was first investigated as the initial state for austenitization and die-quenching by means of SEM-EDS, XRD and micro-XRD. The as-received coating had a thickness of 27.3 ± 3.7 µm. The top surface of the coating (Figure 1a) was covered by a very thin oxide layer enriched in Al and Si, as a result of these materials’ high affinity for oxygen. The matrix of the coating was composed of an Al-Si mixture with a nearly eutectic composition. The silicon particles cannot be clearly distinguished from the α-Al solid solution using a back-scattered electron detector because of the close atomic numbers of Al and Si. However, the regions enriched with Si were clearly visible in the EDS elemental distribution maps (Figure 2), and the presence of Si within the coating was also confirmed by XRD analysis, as was the presence of Al (Figure 3, Si JCPDS card no. 04-016-4861, Al JCPDS card no. 01-072-3440). The needle and platelet-like particles, as well as the continuous layer present at the interface, with an approximate thickness of 3.6 ± 0.6 µm, were formed by the Al-Si-Fe ternary phase.

According to the chemical composition determined by SEM-EDS (Table 2), this ternary phase can be identified as Fe_2_Al_8_Si (also denoted as τ_5_) with a hexagonal structure and the P63/mmc space group [18]. The presence of the τ_5_ phase was also confirmed by XRD and micro-XRD analysis (Figure 3, JCPDS card no 00-020-0030). The irregular shape of the τ_5_/Al-Si coating interface can be explained by the solidification in the Al-Si melt [6,26]. These findings are in good agreement with the literature [4,6,18,20,26]. The purpose of adding Si to the hot-dipping bath is to form this ternary τ_5_ inhibition layer, which impedes the further diffusion of Fe into the coating, and Al in the opposite direction, thus forming a thick layer of intermetallics, especially the Fe_2_Al_5_ brittle intermetallic phase. Furthermore, the addition of Si increases the resistance to both corrosion and elevated temperatures, which is strongly desirable during hot stamping (at temperatures in the 900–930 °C range). Despite the τ_5_ inhibition layer, a thin sublayer reaching thicknesses of 0.7 ± 0.2 µm was formed directly at the steel interface. According to the chemical composition (point 5, Figure 1b), this sublayer was identified as η-Fe_2_Al_5_ with an orthorhombic structure and the Cmcm space group, which has also been mentioned in Refs. [4,5,6,18,20,27,28]. The content of Si (Table 2) in this phase corresponded to that observed by Windmann et al. [6], who reported an even slightly higher content of Si in the η-Fe_2_Al_5_ phase, reaching up to 6.1 at.%.

In addition, a very thin light layer containing elements with higher atomic numbers, such as Si, was observed within the η-Fe_2_Al_5_ phase (marked with a dashed red frame in Figure 1b). However, this phase was below the resolution limits of the EDS detector, and its volume fraction was low, thus it was not detected by XRD either. This phase was also mentioned in the works of other authors [6,20,27] who identified it as Fe_3_Al_2_Si_3_ (further denoted as τ_1_) with a triclinic structure. Its formation was promoted by the addition of Si into the hot dipping bath, and resulted from the low solid solubility of Si in η-Fe_2_Al_5_. Contrary to this, the authors in Refs. [4,5] identified, in addition to the Fe_2_Al_5_ and Fe_2_Al_8_Si phases, a FeAl_3_ phase. The identification was based on the chemical composition determined by SEM-EDS. The presence of FeAl_3_ was also described in Ref. [26], where it formed isolated particles or a continuous layer depending on the dipping time within Fe_2_Al_5_. Note, however, that FeAl_3_ was found after hot-dipping in a bath containing a significantly lower Si content (up to 2%) for an immersion time that was considerably longer than the typical operating time. Shin et al. [18] studied the microstructural evolution of hot-dipped Al-Si coating on 22MnB5 boron steel, and stated that the intermetallic sublayer at the steel substrate interface consisted of Fe_2_Al_5_ and Fe_3_Al_2_Si_3_ when the Si content exceeded 5 wt.%. The authors also provided a TEM analysis of the phase evolution during hot-dip aluminizing. The analyses confirmed the presence of the major τ_5_ phase (hexagonal structure) with grains elongated in the direction of the heat flow and η-Fe_2_Al_5_ (orthorhombic structure), which was separated by a continuous layer of Fe_3_Al_2_Si_3_ (τ_1_) with a triclinic structure. Moreover, the authors observed the presence of a carbon-enriched zone at the steel intermetallic layer interface, which was also discussed in Ref. [26], and identified this layer as the Fe_3_AlC carbide zone.

### 3.2. Chemical and Phase Evolution after Austenitization and Die-Quenching

**Figure 4:**
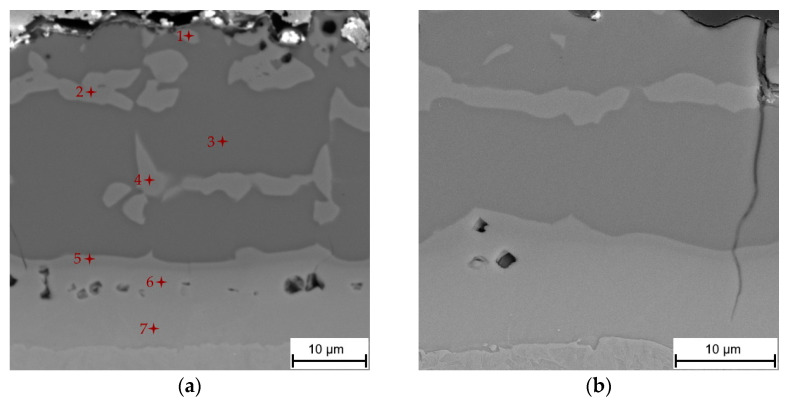
The microstructure of the Al-Si coating on the die-quenched 22MnB5 boron steel: (**a**) cross-section and (**b**) a detailed image of the microcrack reaching into the interdiffusion layer.

The as-received coating with a thickness of 27.3 ± 3.7 µm and the composition of a eutectic mixture of α-Al + Si, Fe_2_Al_8_Si (τ_5_), Fe_2_Al_5_ and Fe_3_Al_2_Si_3_ (τ_1_) phases underwent significant transformation during austenitization and die-quenching. The coating thickness increased to 36.8 ± 3.1 µm, and the coating formed from a multilayered system as a result of the strong diffusion of Fe into the coating, and Al and Si into the steel substrate (Figure 4). The cracks reaching almost to the steel substrate and voids were further present in the coating, which will be discussed later.

**Figure 5:**
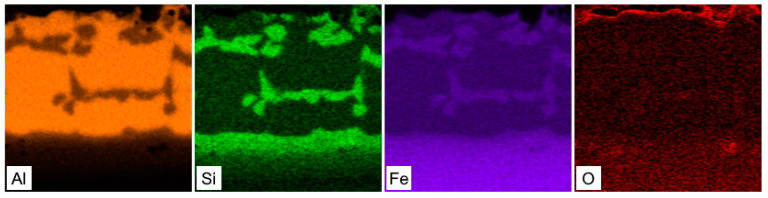
SEM-EDS elemental distribution maps of the Al-Si coating on the die-quenched 22MnB5 boron steel.

The influence of austenitization and die-quenching on the chemical composition and microstructure of the coating was first investigated by SEM-EDS analysis. The chemical composition of each of the structural components found in the layer (marked in Figure 4a) is shown in Table 3. The top-most layer of the coating was enriched with oxygen, as is clearly visible from the SEM-EDS map in Figure 5. The matrix of the coating was attributed to the chemical composition of the Fe_2_Al_5_ phase (point 3, Table 3). The content of Si in the Fe_2_Al_5_ phase was homogeneously distributed in the range of 1.9–2.7 at.%, indicating the relatively poor solubility of Si in this phase with an orthorhombic structure. Other studies [4,20,29], however, identified the matrix of the coating as FeAl_2_, and the presence of both FeAl_2_ and Fe_2_Al_5_ phases was even observed. The predominant formation of the Fe_2_Al_5_ phase can be ascribed to a kinetic factor associated with a highly open structural arrangement of the atoms of this phase. The c-axis of its orthorhombic structure is described to contain only Al atoms, of which about 30% are absent. This large number of vacancies enables a greater diffusion rate of reactant species than in FeAl_2_, and causes directional growth along this c-axis [26,30]. Authors in [26] described the Fe_2_Al_5_ phase as a dominant reaction product at the Fe/Al melt interface following the parabolic growth of this phase in a temperature range of 715–944 °C. The inhibiting effect of Si on the growth of Fe_2_Al_5_ is well known from hot-dip aluminizing. Except for the formation of Al-Si-Fe ternary phases retarding the diffusion of Al and Fe, and thus Fe_2_Al_5_ growth, it was suggested that Si occupies a large number of vacancies on the c-axis and blocks the fast diffusion path of reactant species. Nevertheless, in the present work, the measured Si content was relatively low, reaching from 1.9 to 2.7 at.% [26,31].

The island-like phase (labeled with points 1, 2, and 4 in Figure 4a) contained from 52.1 to 41.4 at.% Al, and 41.5 to 46.6 at.% Fe. According to the chemical composition, this phase was identified as the FeAl(Si) phase. The decreasing content of Al from the top of the coating to the substrate interface and the increasing content of Fe in the opposite manner can be explained by the diffusion path of Al to the steel substrate, and Fe to the coating during austenitization. The measured content of Si in the FeAl(Si) phase was in the range of 5.9–11.1 at.%, which was significantly higher than in Fe_2_Al_5_. The close positions of Al and Si with similar atomic radii allows for the easy substitution of these elements in cubic BCC (B2) crystalline structures [28,32]. Similar contents of Si in the FeAl(Si) phase were also observed in Refs. [6,21]. The higher solid solubility of Si and the Fe-enrichment of the coating by the diffusion of Al towards the steel substrate, and Fe into the coating, with elongating dwell time were stated as the reasons for the FeAl(Si) phase’s formation in Refs. [6,17]. Cho et al. [24] noted that the island-like phase, in addition to the FeAl phase, was formed by an adjacent Fe_x_Al_y_Si_z_ ternary phase that was not identified due to its fine nature and probably low symmetry.

**Figure 6:**
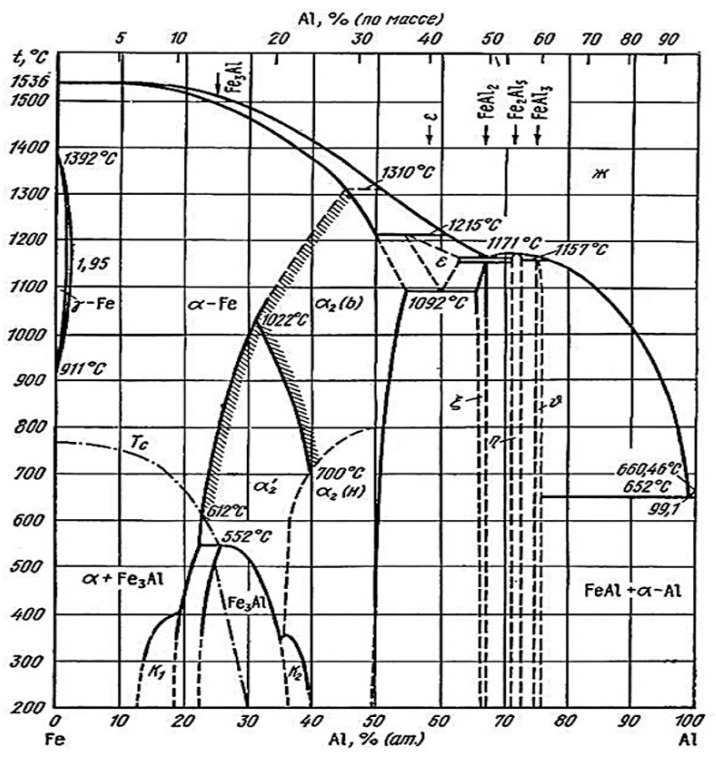
Fe-Al Binary phase diagram [33].

A relatively thick layer, which was also described as an interdiffusion layer with an average thickness of 11.5 ± 1.0 µm, was formed at the substrate/coating interface with two distinguishable sublayers. The first sublayer on the top (labeled with point 5 in Figure 4a) had a chemical composition similar to the compositions at points 1, 2 and 4, which corresponded to the presence of the FeAl(Si) phase. Its occurrence at the Fe_2_Al_5_ interdiffusion layer interface can be explained by the binary Al-Fe phase diagram (Figure 6). The diffusion of Fe towards the coating and of Al to the steel substrate led to the Fe-enrichment of the interface, resulting in the transformation of Fe_2_Al_5_ to the FeAl(Si) iron-richer phase. The sublayer closer to the steel interface (labeled with points 6 and 7 in Figure 4a) is described in the majority of the literature [6,17,20,21,22,29] as an α-Fe(Al,Si) solid solution, which is reasonable, since Al and Si are strong ferrite stabilizers that diffuse to the steel substrate during austenitization. However, some studies [5,23] identified this layer as Fe_3_Al, which has also been reported by others [4,32], wherein the authors described the phase transformation of high-temperature α-Fe(Al, Si) to the low-temperature stable Fe_3_Al (D0_3_) ordered phase. In the present study, the concentration gradient of Al and Si in this sublayer (α-Fe(Al, Si)/Fe_3_Al) was noticed, which is shown in the SEM-EDS map in Figure 5, and the chemical composition is shown in Table 3. Furthermore, the Si-enriched area was observed within the top sublayer of the interdiffusion layer (point 5) extending further towards the steel, which is in accordance with Ref. [6]. Nevertheless, we cannot clearly confirm whether both phases (α-Fe and Fe_3_Al) are present based solely on the chemical composition. The micro-XRD analysis revealed the presence of Fe_2_Al_5_; however, other phases were not detected by micro-XRD or XRD analysis due to the large thickness of the coating and due to the relatively low volume fraction of the phases.

As previously mentioned, the voids present in the coating were located in the interdiffusion layer and beneath the top surface of the coating. The mechanism of void formation in the interdiffusion layer is correlated with the Kirkendall effect [4,6,20,29]. During the first few minutes of austenitization, the Fe diffusion is more pronounced, which is supported by the Al-Si coating melting at its eutectic temperature (575 °C) [6,20,26,34]. Subsequently, Fe enrichment leads to the solidification of the partially melted material, allowing Al-Fe-Si intermetallics to form with higher melting temperatures, especially the Fe_2_Al_5_ phase, because of the extremely rapid growth along the c-axis compared to other phases [5,17]. Therefore, the further diffusion of Fe, Al and Si atoms occurs in the solid state, where the diffusion of Al atoms becomes more active towards the steel substrate. The higher diffusion rate of Al can be attributed to the high mobility of Al atoms along the c-axis of the orthorhombic Fe_2_Al_5_ phase with high vacancy concentration, which is responsible for the void formation [26,35]. This corroborates Windmann et al.’s results [6], as they observed the formation of voids in a fully solidified coating composed of Al-Fe-Si intermetallic phases as well as their increasing volume fraction, which accompanied the increasing austenitization temperature. The authors also observed that the diffusion coefficients of aluminum in the Al-Fe-rich intermetallic phases formed in the coating were larger than those of Fe. The faster self-diffusion of Al than Fe in three different Fe-Al alloys (25.5 at.%, 33.0 at.%, 48.0 at.% Al) was also described in Ref. [36]. Therefore, it might be concluded that the Kirkendall voids are caused by the diffusion of Al into the steel substrate, being more pronounced than Fe diffusion in the opposite direction, considering diffusion in the solid state. The voids beneath the top surface of the coating were probably caused by the diffusion of Al to form Al_2_O_3_, and the reaction with the furnace atmosphere [4].

#### TEM Characterization and Coating Phase Identification

**Figure 7:**
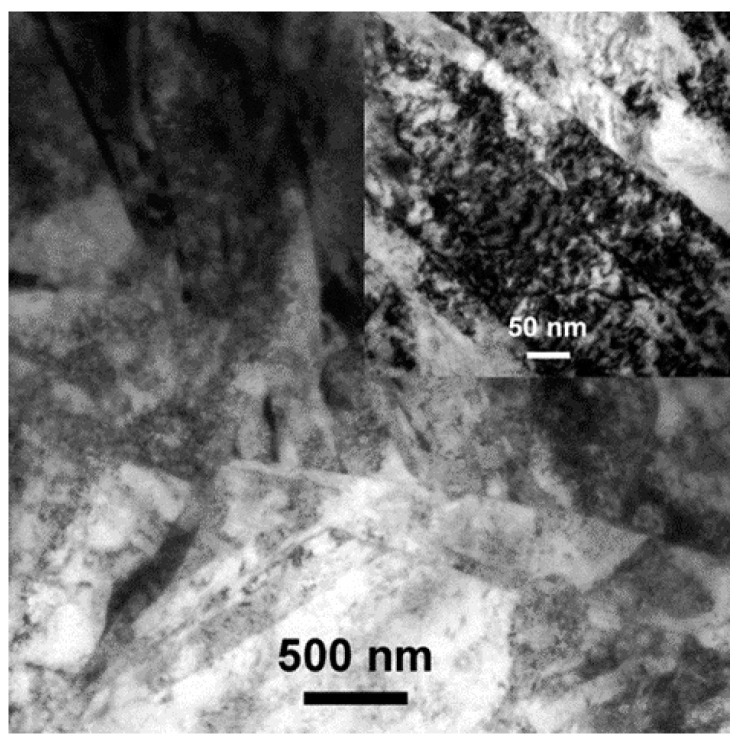
TEM micrograph of typical martensite laths of the hardened 22MnB5.

All the different Al-Si coating layers were identified and then characterized by TEM. In this regard, at least four chemically different zones were already identified by scanning electron microscopy (Figure 4). As such, TEM layer characterization was carried out starting from the hardened martensite structure of the 22MnB5 steel, and then proceeding through the Al-Si coating, ending at the outermost coating zones. Figure 7 reports a representative bright-field (BF) image showing the typical martensitic structure of the coated steel. This was mainly composed of martensite laths with 70 ± 20 nm thicknesses (Figure 7 inset).

**Figure 8:**
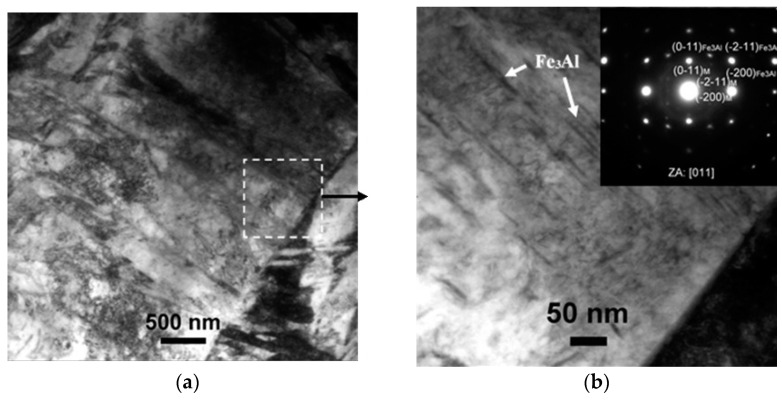
TEM micrographs of (**a**) the martensite structure in direct contact with the coating, (**b**) where some Fe_3_Al precipitates were detected. Inset in (**b**) is the indexed CB-SAEDP of the Fe_3_Al phase (indicated by the arrow in the micrograph).

The martensite directly in contact with the coating revealed the presence of some Al-bearing rod-shaped particles whose stoichiometry was fully compatible with Fe_3_Al. This was determined by CB-SAEDP, and the related representative image and diffraction pattern are reported in Figure 8. The very fine nature of the Fe_3_Al rod-shaped particles can be clarified with a closer look at the Fe-Al phase diagram (Figure 6). The Fe_3_Al phase is stable in a concentration range from 22.5 at.% to 36.5 at.% Al and transformed to FeAl or α-Fe at temperatures above 545 °C [24]. Considering that the typical temperature is 800–850 °C at the beginning of hot stamping and the die-quenching in the tool is maintained usually for 8–12 s [37], there is not enough time for transformation and phase growth, resulting in the very fine nature of the rod-shaped Fe_3_Al particles. The transformation of α-Fe to Fe_3_Al was described in Ref. [32] after austenitization at 1050 °C, deformation and cooling. Furthermore, the gradual transition from the bcc crystal structure of α-Fe to D0_3_ of Fe_3_Al and to B2 of FeAl at the substrate/coating interface was reported by Cho et al. [24]. However, the authors did not observe any evidence of interfaces between these three different crystal structures.

**Figure 9:**
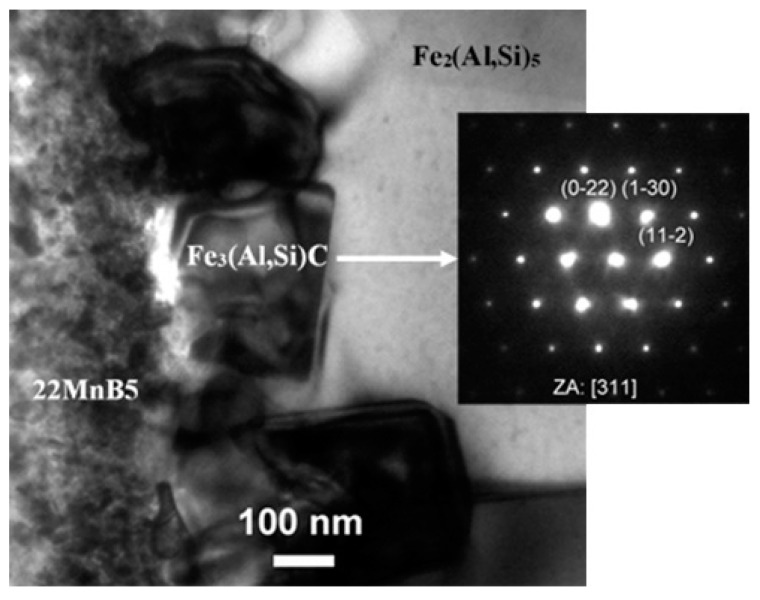
TEM micrograph of the first coating sublayer directly in contact with the martensite structure of the hardened 22MnB5 steel. Inset is the indexed CB-SAEDP, revealing the crystallographic nature of this thin sublayer identified as Fe_3_(Al,Si)C.

**Figure 10:**
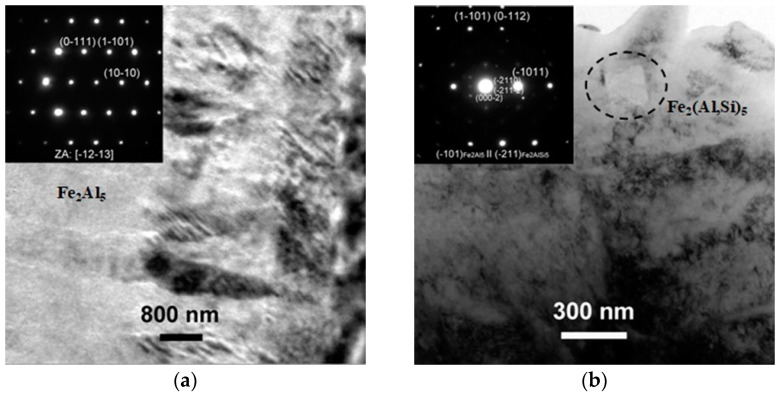
TEM micrographs of (**a**) the coating layer composed of Fe_2_Al_5_ and (**b**) detail of the Fe_2_Al_5_ layer, showing a cuboid-like Fe_2_(Al,Si)_5_ particle.

The coating layer immediately in contact with the steel was mainly composed of a thin layer of Fe_3_(Al,Si)C, which featured a fine nanometric granular morphology with a mean thickness of 180 ± 90 nm. This layer was not always continuously distributed, and its thickness was quite scattered, ranging from a minimum of almost null to a maximum of up to 340 nm. A representative image of this thin layer is reported in Figure 9 along with the related SAEDP (inset). This carbide phase was identified as a product of solid-state interdiffusion between low carbon steel and pure Al at 600 °C in Ref. [30], and was further found at the interface of the 22MnB5 steel and Al-Si hot-dipped coating in Ref. [18]. This layer is probably formed by the inability of carbon to diffuse through the Fe-Al intermetallic phases, and carbon becomes concentrated at the steel interface where it forms ternary carbides [26]. These carbides seem to remain unchanged after austenitization and die-quenching. Further away from the thin Fe_3_(Al,Si)C layer, another thin layer was detected. This was more compact and continuous than the former layer. This second layer was found to be composed of Fe_2_(Al,Si)_5_ with a thickness of 350 ± 30 nm. Further away from the 22MnB5 steel, the Al-Si coating was formed by a thicker layer of Fe_2_Al_5_. Within this layer, some nanometric particles of Fe_2_(Al,Si)_5_ were also detected. These particles were fairly scattered and cuboid-shaped. Both the coating layer and nanoparticles are shown in the TEM image of Figure 10. In particular, the inset SAEDP of Figure 10b clearly shows that there is a crystallographic correlation between the small cuboid-like Fe_2_(Al,Si)_5_ particles and the surrounding layer of Fe_2_Al_5_; this was determined to be (-211)Fe_2_(Al,Si)_5_//(-101)Fe_2_Al_5_.

**Figure 11:**
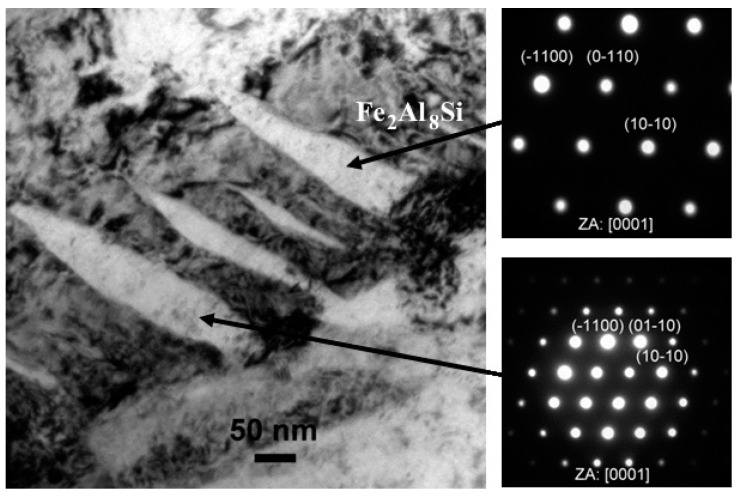
TEM micrograph of the interface between the Fe_2_Al_5_ layer and the outermost coating layer. Lenticular-shaped zones were identified by SAEDPs (insets) as Fe_2_Al_8_Si.

Further away, the coating changed its composition again, and this layer had the same chemical composition and crystallographic structure as the innermost Fe_2_(Al,Si)_5_ layer, which was followed by a thin layer of Fe_2_Al_5_. This latter layer was followed by the outermost coating layer with a chemical composition of Fe_2_Al_8_Si. This was indeed a fairly scattered layer with a nonuniform thickness. Figure 11 shows a representative image of the interface between Fe_2_Al_5_ and Fe_2_Al_8_Si. SAEDPs were recorded and are also reported in Figure 11 insets. The Fe_2_Al_8_Si ternary phase is a major reaction product formed at the interface of the steel and Al-Si hot-dipped coating, as well as partly dispersed in the Al-Si coating matrix. The presence of this ternary phase in the outermost coating layer can be explained by the gradual enrichment of the coating with Fe and vice versa by the diffusion of Al and Si into the steel substrate. As a result of this Fe enrichment, the ternary phase was shifted towards the coating surface, where it formed a discontinuous layer due to its partial conversion to the Fe richer phases. Windmann et al. [17] reported the occurrence of Fe_2_Al_8_Si after 1 min of austenitization at 920 °C, and its transformation to an iron-richer phase of type Fe_2_Al_5_ with increasing dwell time. However, the Fe_2_Al_8_Si ternary phase was clearly identified in the coating, even after an 8 min dwell time at 920 °C in the present study.

### 3.3. Changes in the Surface Condition after Die-quenching

#### 3.3.1. XPS Analysis of the Surface Condition

**Figure 12:**
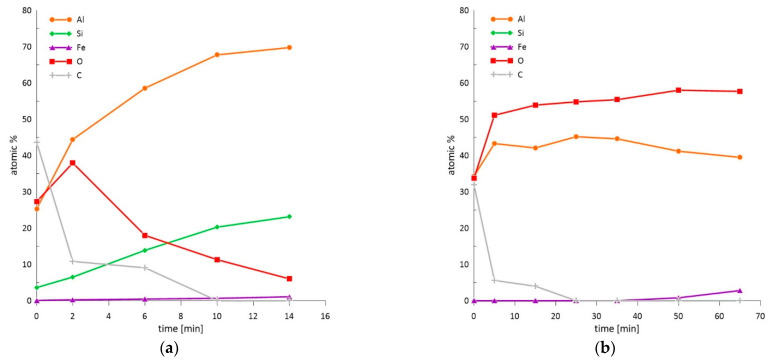
XPS depth profiles of (**a**) the chemical composition of the as-received 22MnB5 boron steel in the hot-dipped condition and (**b**) the die-quenched 22MnB5 boron steel.

**Figure 13:**
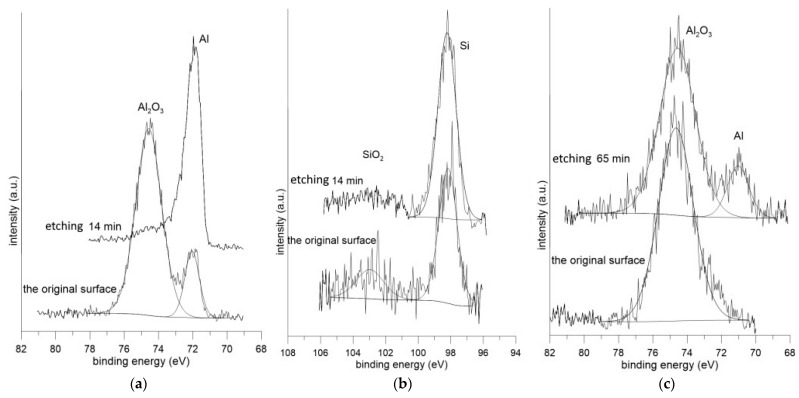
XPS spectra of (**a**,**b**) the Al-Si hot-dipped coating on as-received 22MnB5 boron steel and (**c**) the Al-Si coating on the die-quenched 22MnB5 boron steel.

Since SEM-EDS analysis can be used to analyze the deeper parts of materials, the exact nature of the present oxidic products was further examined by obtaining a depth profile of the chemical composition measured by X-ray photoelectron spectroscopy (Figure 12). The concentration dependence is plotted over time, since the etching speed is not constant over time. From the initial oxide layer of the as-received sample with a thickness of 14 nm (Figure 12a), the austenitization led to the strong enrichment of the surface with oxygen, creating an oxide layer with a total thickness of 76 nm (Figure 12b). The oxides detected on the surface of the as-received sample consisted of Al_2_O_3_, SiO_2_, and Al_2_O_3_ after austenitization and die-quenching (Figure 13). In industrial conditions, the whole austenitization process and transferral to the press do not occur under a protective atmosphere, as was the case in the experiment in the present work. As such, the coating meets the air at high temperatures in the order of minutes. The high affinity of aluminum to oxygen, as well as the elevated temperature, cause the diffusion of Al to the coating surface and significant oxygen enrichment due to the formation of a protective Al_2_O_3_ layer, which further protects the coating itself and the steel substrate from scaling and decarburization [4,37]. However, the thick oxide layer influences the contact resistance during resistance spot welding in the automotive industry. Moreover, the presence of oxides at the surface can lead to inhomogeneous current flow and violent heat generation [11,38].

#### 3.3.2. Roughness Measurement of the Surface

In contrast to the smooth and uniform features characteristic of the hot-dipped condition (Figure 1), the occurrence of voids and microcracks accompanies phase transformations in the coating and steel during austenitization and die-quenching (Figure 4). The microcracks nucleated at the top of the coating as a spot of increased stress concentration and then propagated through the coating [29]. Note that these microcracks were intercepted within the interdiffusion layer and did not reach the steel substrate. The interdiffusion layer was rich in iron (75.9 to 90.8 at.%) and tended to show a ductile behavior [20,29,32].

A reasonable explanation for the microcrack’s formation is the thermal stress induced during austenitization and rapid cooling. The phase development during this heat cycle is connected both with the volume changes and with differences in the thermal expansion coefficients of the newly formed phases, resulting in thermal stresses. Gui et al. [20] stated that the densities of the main intermetallic phase Fe_2_Al_8_Si (τ_5_) at the steel/coating interface under the hot-dipped condition were 3.62 g/cm^3^ and 4.11 g/cm^3^ for Fe_2_Al_5_ and 5.37 g/cm^3^ for the FeAl phases. Both the abovementioned phases (Fe_2_Al_5_ and FeAl) were also present in the die-quenched condition. The density of Fe is generally known to be 7.7 g/cm^3^. Ruan et al. [39] studied the thermal expansion coefficient of the Al-Fe system (10–75 at.% Al) by a dilatometric method. They observed an increase in the thermal expansion coefficient at the same temperature and sensitivity to temperature with increasing the Fe content from 1.62 × 10^−5^ K^−1^ (Al75Fe25) to 2.23 × 10^−5^ K^−1^ (Al30Fe70), and then decreasing to 1.41 × 10^−5^ K^−1^ (Al10Fe90). Moreover, Fe_2_Al_5_ is known to be fragile with a low fracture toughness (1.2 MPa·m^1/2^), which promotes microcrack growth and propagation [20,29]. When the maximum strength of the coating is exceeded by the action of thermal stresses and volume changes, the microcracks nucleate and easily grow in the fragile coating. These microcracks then may become wide and can intersect the voids in the coating, leading to coating delamination during die-quenching. More importantly, areas with exposed substrate behave as potential weak spots for corrosion [29].

**Figure 14:**
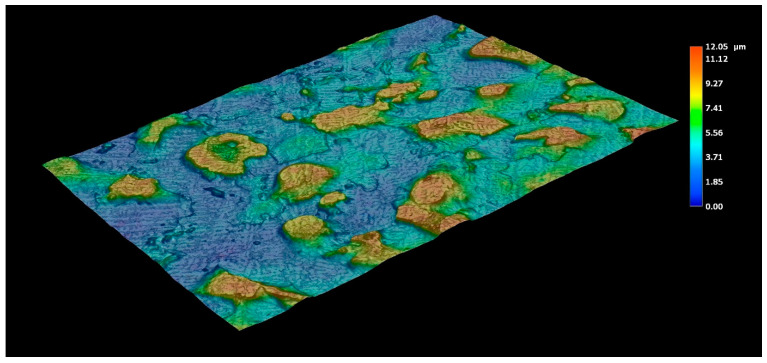
The topography profile of the Al-Si hot-dipped coating on as-received 22MnB5 boron steel.

**Figure 15:**
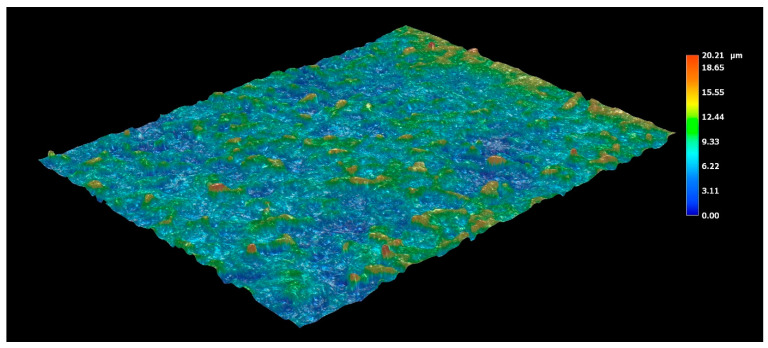
The topography profile of the Al-Si coating on the die-quenched 22MnB5 boron steel.

In addition to crack occurrence in the coating, the volume changes also resulted in a change in surface topography. The surface profile and roughness were measured by a Keyence digital microscope and roughness tester using three linescans. The topography profile of the as-received sample with a hot-dipped coating is shown in Figure 14. The roughness parameters R_a_ and R_pc_ were 1.88 µm and 53 peaks/cm, respectively, and both values confirmed that the surface was relatively smooth. Austenitization and die-quenching caused the roughness parameters to increase to R_a_ = 2.50 µm and R_pc_ = 142 peaks/cm (Figure 15). The hot-dipped Al-Si coating melts during austenitization and resolidifies again due to Fe enrichment, leading to the formation of Al-Fe-Si intermediate phases. These phases were inhomogeneously distributed and, as was described above, have different volumes and thermal expansion coefficients. It can be assumed that some of the Al-Fe-Si phases were exposed to the surface, or their formation allowed the surface roughness to increase. In addition, the diffusion-related transport of atoms might also contribute to the formation of shallow valleys, as shown in Figure 15. This presumption also corresponds to the dimensions of these phases, which have already been shown in Figure 4.

## 4. Conclusions

It was found that the initial hot-dipped Al-Si coating on commercially available 22MnB5 steel was composed of Al, Si matrix, FeAl_8_Si_2_ (τ_5_) phase and two thin sublayers of η-Fe_2_Al_5_ and Fe_3_Al_2_Si_3_ (τ_1_) at the coating/substrate interface. Heat treatment at 920 °C for 8 min followed by die-quenching resulted in strong Fe diffusion to the coating, changing the phase composition and causing an increase in the thickness. In addition to the phases FeAl(Si) and Fe_2_Al_5_, and the interdiffusion layer forming the multilayered structure of the coating, electron diffraction revealed layers and phases of a very fine nature. The thin Fe_3_(Al,Si)C coating layer (180 ± 90 nm), detected by other authors after hot-dipping, was found at the site of contact with the steel. However, the present layer was retained even after heat treatment, which has not been reported in the works of others. Al-bearing rod-shaped particles in the immediate vicinity of the steel, determined as Fe_3_Al, were the result of rapid cooling during die-quenching from the austenitization temperature. The presence of an Fe_2_Al_5_ phase further away from the steel substrate (matrix of the coating) was confirmed by electron diffraction, and cuboid-shaped nanometric particles of Fe_2_(Al,Si)_5_ were detected within this phase. The residues of Fe_2_Al_8_Si phase in the outermost coating layer can be attributed to the shift of this phase due to the significant enrichment of the coating with Fe, and to its partial conversion to Fe-richer phases.

Furthermore, the surface of the Al-Si coating was covered by the oxide layer initially formed by Al_2_O_3_ and SiO_2_, the thickness of which increased during austenitization and die-quenching from 14 to 76 nm. After this heat treatment, the layer was composed mostly of an Al_2_O_3_.

The above-described phase development during the heat treatment, connected both with the volume changes and with differences in the thermal expansion coefficients of the newly formed phases, resulted in a surface roughness increase from 1.88 to 2.50 µm (in terms of R_a_ value), and to the microcracks formation.

## Figures and Tables

**Table 1:** The chemical composition of 22MnB5 steel without the Al-Si coating, as measured by OES.

Element	C	Si	Mn	P	S	Al	Cr	Ti	B
wt.%	0.22	0.24	1.17	0.010	0.002	0.044	0.19	0.026	0.0027

**Table 2:** The results from the SEM-EDS point analysis, presented as atomic percentages, and the corresponding phase composition.

Element	O	Al	Si	Cr	Mn	Fe	Phase
point 1	1.9	79.4	17.6	-	-	1.1	Al + Si, oxides
point 2	-	89.4	10.2	-	-	0.4	α-Al + Si
point 3	-	71.5	11.3	-	-	17.2	τ_5_-Fe_2_Al_8_Si
point 4	-	67.8	11.8	-	0.2	20.2	τ_5_-Fe_2_Al_8_Si
point 5	-	58.9	5.7	0.1	0.4	34.9	Fe_2_Al_5_
point 6	-	-	0.5	0.3	1.3	97.9	Steel

**Table 3:** The results from the SEM-EDS point analysis, presented as atomic percentages, and the given phase composition.

Element	Al	Si	Cr	Mn	Fe	Phase
point 1	52.1	5.9	-	0.5	41.5	FeAl(Si)
point 2	46.2	10.0	0.2	0.8	42.8	FeAl(Si)
point 3	68.0	1.9	-	0.3	29.8	Fe_2_Al_5_
point 4	41.4	11.1	-	0.9	46.6	FeAl(Si)
point 5	39.7	10.6	0.1	0.7	48.9	FeAl(Si)
point 6	16.5	6.6	0.2	0.8	75.9	α-Fe(Al,Si)
point 7	7.9	-	0.2	1.1	90.8	α-Fe(Al,Si)

## Data Availability

Data is contained within the article.
